# Application of ultrasound in the management of TRASH (the radiographic appearance seemed harmless) fractures in preschool children: A review

**DOI:** 10.1097/MD.0000000000034855

**Published:** 2023-08-25

**Authors:** Guoqiang Jia, Jun Sun

**Affiliations:** a Department of Orthopaedics, Provincial Children’s Hospital of Anhui Medical University, Hefei, Anhui Province, China.

**Keywords:** fracture, preschool, TRASH, ultrasound

## Abstract

Elbow fracture is one of the most common fractures in preschool children, and the secondary ossification center appears regularly with age increasing. Transphyseal separation of the distal humerus, medial humeral condyle fracture, lateral humeral condyle fracture, radial head osteochondral separation, and Monteggia fracture (minimal ulnar bow type) are difficult to diagnose based on X-ray films alone because of the unossified secondary ossification center or a suspicious non-alignment of the anatomical cartilage of the elbow joint in preschool children. These 5 fractures above are defined as The Radiographic Appearance Seemed Harmless fractures in preschool children (TRASH-PS). The TRASH-PS fractures must be taken into consideration when there is swelling at the injured site without fracture feature on X-ray. Additionally, relevant misdiagnosis or unsuitable management can lead to elbow dysfunction and deformity. Therefore, this work reviews the application of ultrasound in the management of TRASH-PS fractures.

## 1. Introduction

The elbow joint of children contains a large amount of cartilage and presents a dynamic ossification of the secondary ossification center with growth. Ossification of the elbow proceeds at a predictable rate and the order of ossification is from capitellum of humerus, radial head, medial epicondyle, olecranon of ulna and trochlea of humerus, to lateral epicondyle latest on X-ray. The nature of preschool children with unossified secondary ossification center is seen to be normal-like on the X-ray images, and the large cartilaginous “empty spaces” may harbor harmful injuries, which could cause serious consequences or deformities.^[1–3]^

In 2010, Waters et al identified 8 fractures with Radiographic Appearance Seemed Harmless (TRASH) fracture of the elbow, recommending that surgeons should be more careful to do physical examination.^[4]^ The ultrasound or magnetic resonance imaging were also advised to confirm diagnoses.^[5–8]^ Due to the high miss-diagnose rate in TRASH preschool patients (TRASH-PS), we reviewed the meaningful use of ultrasound of TRASH-PS fractures.

## 2. Methods

### 2.1. Ethics approval and consent to participate

Written informed consent is obtained from legal guardian of participants.

The review was conducted according to the guidelines of the Declaration of Helsinki, and approved by the Institutional Review Board of Children Hospital of Fudan University Anhui Hospital (EYLL-2022-081).

**Figure 1.**
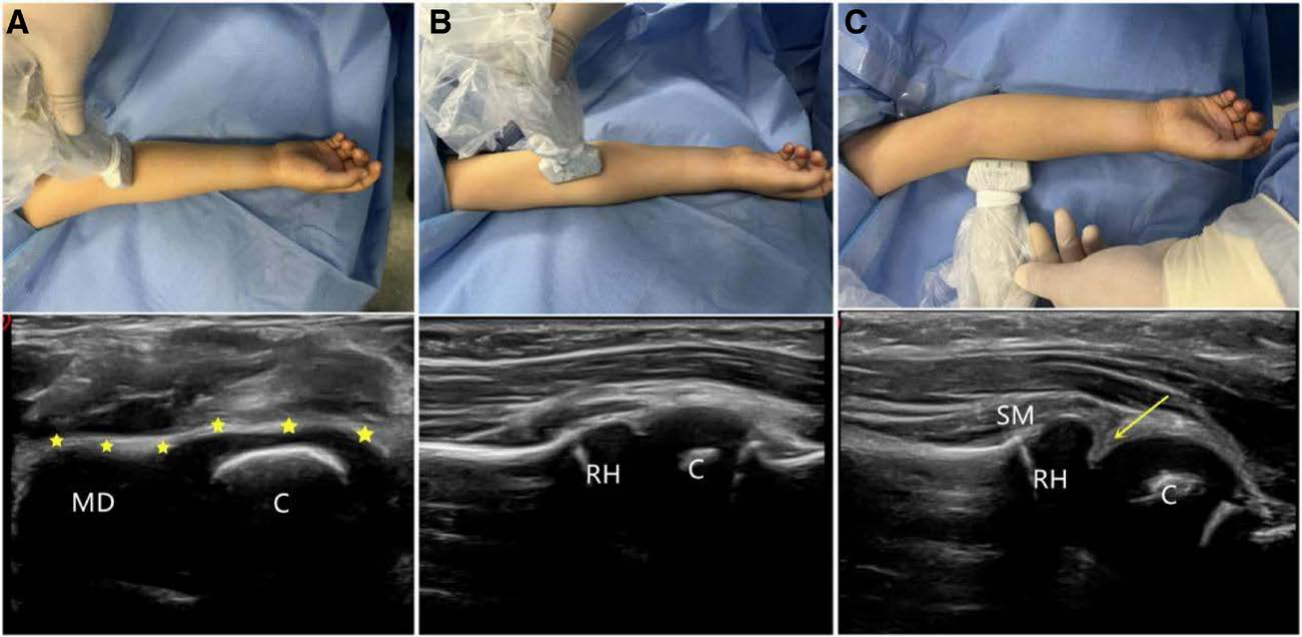
These are pictures of the ultrasound examination positions and mapping related scans of a 4-yr-old boy. (A) On the transverse scan, the normal articular cartilage of distal humeral was clearly showed as a continuous, black, and waving shape (yellow stars). (B) On the coronal longitudinal scan, it showed that there were normal “double-hump sign” and “congruency sign” in radio-capitellum joint. (C) The sagittal longitudinal scan of ultrasound showed that the supinator muscle was advanced and annular ligament was entrapped, indicating a “hook sign” (yellow arrow). C = capitellum; MD = medial condylar; RH = radial head; SM = supinator muscle.

The ultrasound examination has some advantages, such as absence of ionizing radiation, dynamic monitoring in multiple directions of elbow, and displaying articular cartilage clear. Although the use of ultrasound is operator-dependent, it is still promising in clinics. Regarding our daily practice, the ultrasound examination was executed by 1 properly trained orthopedist with high-frequency (15–20 Mhz) linear probes (L: 15-4, Wisonic Ultrasonic Equipment Co., Ltd, Shenzhen, China) in a musculoskeletal mode. Three standardized sonographic planes (one transverse and 2 longitudinal scans) were performed to detect TRASH-PS fractures. On transverse scan, the articular cartilage of distal humeral was clearly displaced as a continuous black cartilaginous components (Fig. 1A). On longitudinal scans, the radial neck, radio-capitellum alignment, and annular ligament were displaced well. There were 2 normal signs, which were “double-hump sign” and “congruency sign” in coronal longitudinal scan (Fig. 1B). The sagittal longitudinal scan would evaluate the supinator muscle and annular ligament, and a “hook sign” indicated annular ligament was advanced and entrapped (Fig. 1C).

## 3. Discussions

### 3.1. Characteristics and application of ultrasound in TRASH-PS fractures

#### 3.1.1. Transphyseal separation of the distal humerus.

**Figure 2.**
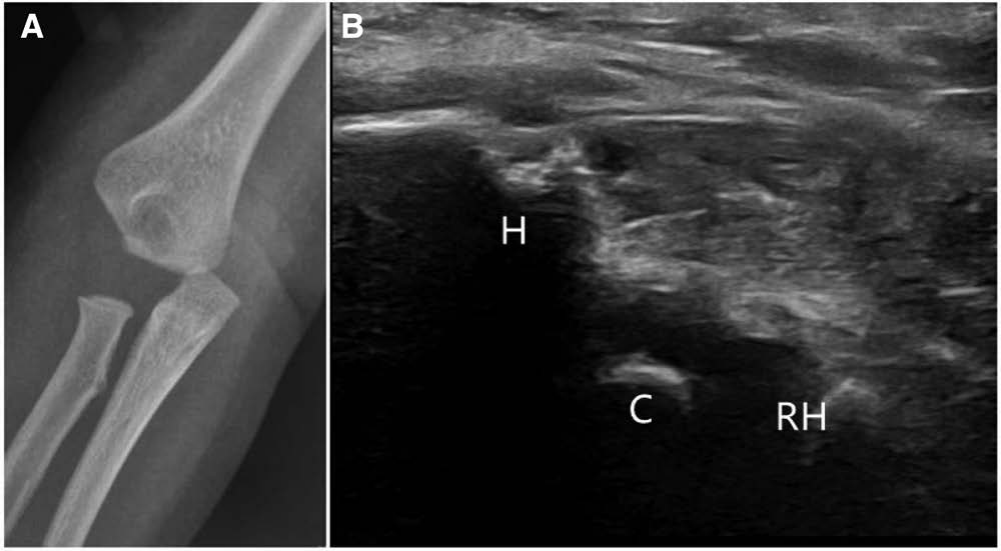
A 1-yr-old boy was diagnosed with a transphyseal separation of the distal humerus. (A) X-ray showed a dislocation-like fracture of the elbow joint. (B) The coronal longitudinal scan of ultrasound showed that there was a normal alignment in radio-capitellum joint and the capitellum was separated from distal humer. C = capitellum; H = humer, RH = radial head.

The secondary ossification center of the capitellum humeri appears at ages 0.5 to 1 year, and humeral epiphyseal separation fractures are observed mainly in infants or toddlers. The general standards for elbow fracture diagnosis include the Baumann angle, lateral humeral line, and humerocondylar angle on X-ray films.^[9–11]^ On radiographs, the transphyseal separation of the distal humerus appears as dislocation-like (Salter-Harris type I) (Fig. 2) or dislocation-like with a metaphyseal fragment slice (Salter-Harris type II) as described by Delee.^[12]^ Due to the un-ossification of cartilage, the transphyseal separation of the distal humerus is easily to be misdiagnosed as an elbow dislocation. For Salter-Harris type II, it is often confused with type IV humeral lateral condylar fracture.^[13]^ The displacement of transphyseal distal humeral separation is mostly posteromedial while the direction of elbow dislocation secondary to displaced lateral condyle fracture is posterolateral. In addition, the radio-capitellum alignment in transphyseal fracture is intact; however, there is usually radio-capitellum alignment subluxation in type IV humeral lateral condyle fractures.

#### 3.1.2. Medial condyle fracture of the humerus.

**Figure 3.**
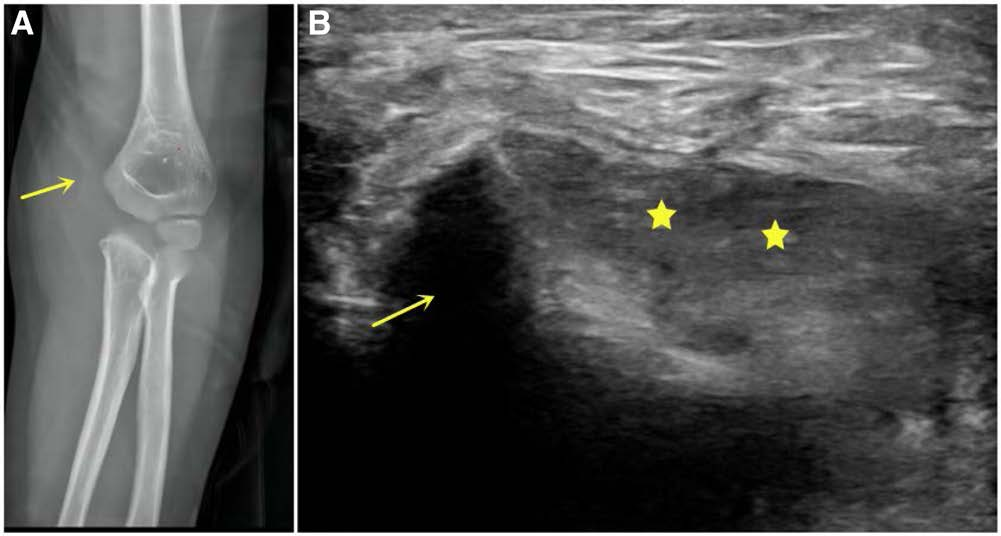
(A) A 5-yr-old boy, X-ray image showed swelling of the medial soft tissue and a thin “C” shaped fracture fragment (yellow arrow) was visible. (B) The transverse ultrasound scan showed that the cartilage of medial condyle was displaced (yellow arrow) from cartilage hinge of distal humer (yellow star).

The secondary ossification center of the medial humeral condyle appears at approximately 8 to 9 years. The fracture of the medial condyle of the humerus is also a common TRASH-PS lesion. Generally, there are 2 typical manifestations on X-ray films. Type I is that only the medial soft tissue is highly swollen with no signs of fracture fragment. This type indicates a pure medial condyle cartilage fracture of the humerus. Type II is visible on the anteroposterior X-ray film, where an inverted wafer “C”-shaped fracture fragment is present inside the medial soft tissue. The “C” sign is a special feature of X-ray images of the medial condyle fracture (Fig. 3A), and the age of type II patients is usually older than the one of type I. Encountered with pain, ecchymosis, swelling over the medial side, and a metaphyseal wafer of bone within the medial aspect of the elbow in X-ray, the suspicion of medial condylar fracture should arise and further imaging techniques like ultrasound should be performed (Fig. 3B).^[14]^

#### 3.1.3. Humeral lateral condyle fracture.

**Figure 4.**
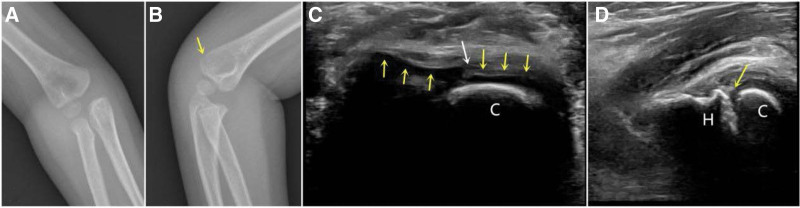
(A) A 4-yr-old boy, a slightly avulsion fracture of the lateral condylar of humerus can be seen on the anteroposterior film (arrow). (B) A slightly avulsion fracture was latent on the lateral film. (C) The transverse ultrasound showed the accumulated articular cartilage gap (white arrow) and step-off (yellow arrow) of the fracture. (D) The longitudinal ultrasound showed the fracture gap (yellow arrow) was much clearer on ultrasound than the ones in X-ray. C = capitellum, H = humer.

The minimally displaced humeral lateral condyle avulsion shear fracture is another TRASH-PS lesion and the treatment is easily delayed in developing countries. The main reasons are as follows: the first is a missed diagnosis because an internal oblique film is needed which is unusually used firstly in an emergency. However, ultrasound could detect fracture gap and step-off of cartilage hinge clearly than X-ray, and the displacement was viewed between capitellum and humer (Fig. 4). The second reason is the high re-displacement rate of minimally displaced humeral lateral condyle fracture.^[15]^ Therefore, ultrasound is considered a useful tool.^[16,17]^ Humerus lateral condyle fractures in young children also require a differential diagnosis from that of a distal humerus transphyseal separation with lateral metaphyseal fragmentation.^[18]^

#### 3.1.4. Radial head osteochondral separation.

**Figure 5.**
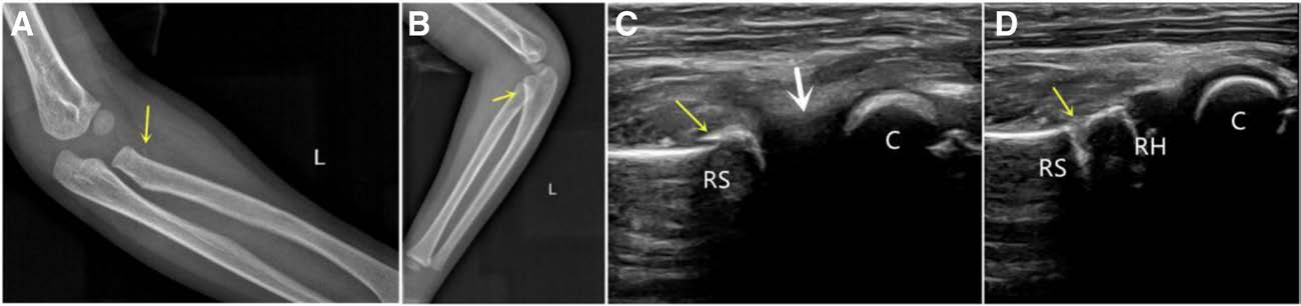
(A) A 5-yr-old boy, the displacement fragment was barely visible (yellow arrow) on the anteroposterior radiograph film. (B) There was an overlapping fracture shadow on the lateral film behind the proximal ulna (yellow arrow). (C) The sagittal longitudinal ultrasound scan showed obvious displacement of the radial head and a metaphyseal fracture line (yellow arrow), but the radial head was disappeared (white arrow). (D) Ultrasound showed normal “double-hump sign” and “congruency sign” after closed reduction. C = capitellum, RH = radial head, RS = radial shaft.

The ossification of the radial head occurs at the ages of 5 to 6 years, and the displaced radial head cannot be detected on X-ray until ossified, especially in Salter-Harris I. Usually, only a fracture fragment is visible along the radial axis at the proximal end of the radius on the anteroposterior film in Salter-Harris II. The fracture fragment may not be visible in the lateral film due to the shielding of the proximal part of the ulna, or an overlapping image of the fracture can be vaguely seen.^[6,7]^ If the patient is rejected to rotate the swollen forearm, ultrasound is a suitable evaluation tool to avoid misdiagnosis (Fig. 5). Ultrasound monitoring is recommended to assist in reduction because it facilitates the evaluation of the radio-capitellum alignment dynamically (Fig. 5).^[18]^

#### 3.1.5. Monteggia fracture type I.

**Figure 6.**
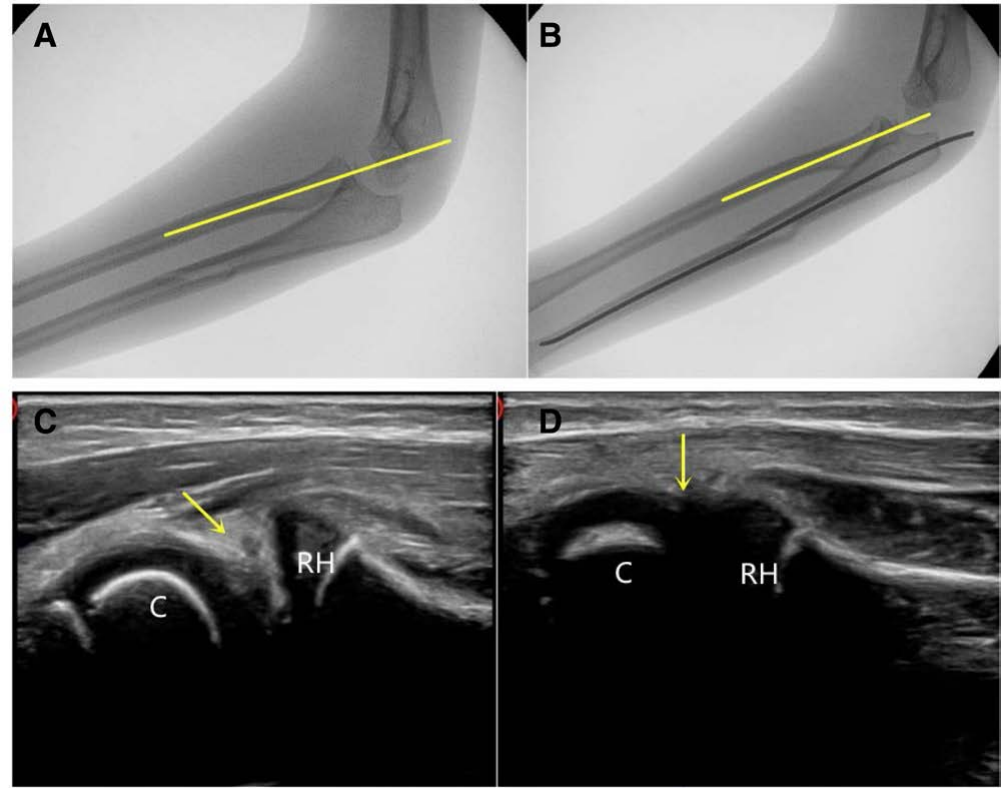
(A) A 5-yr-old girl, the fracture of the ulna was displaced on the X-ray lateral view, and the axis of the radial neck passed through the upper 1/3 of the capitellum (arrow). (B) The axis of the radial neck passed through the middle 1/3 of the capitellum after ultrasound monitoring closed reduction. (C) Ultrasound showed obvious displacement of the radial head with soft tissue incarceration (arrow) before reduction. (D) Ultrasound showed normal “double-hump sign” and “congruency sign” after closed reduction (arrow). C = capitellum, RH = radial head.

The Monteggia fracture type I of Bado classification is also an easily misdiagnosed TRASH-PS lesion, especially the ones with subluxated radio-capitellum alignment combination with the minimal ulnar bow sign. In these cases, the radial axis often passes through the upper 1/3 of the capitellum. On magnetic resonance imaging, the annular ligament is usually entrapped in the radio-capitellum joint.^[19]^ Clinically, the diagnostic standards of Monteggia fracture are the humeral lateral line on the anteroposterior plate and the long axis of radial shaft on the lateral plate.^[20,21]^ On ultrasound, the subluxation is obvious and there is usually annular ligament incarceration in radio-capitellum joint, the normal “double-hump sign” and “congruency sign” disappeared (Fig. 6). On the lateral longitudinal scan of the elbow via ultrasound, it could be suspicious Monteggia fracture when the long axis of radial shaft fails to pass through the middle third of the capitellum in any degree of elbow flexion.^[19]^

### 3.2. Useful strategies for the management of TRASH-PS fractures

Currently, common strategies used to evaluate TRASH-PS fractures during patient examinations are as follows: (a) careful physical examination. Surgeons should pay more attention to the responses of children during physical examination to confirm the painful site and (b) Ultrasound examination is recommended to prevent missed diagnosis.^[22]^ Ultrasound is recommended for a slightly displaced lateral condylar fracture of the humerus to determine whether surgical treatment is required according to the damage degree of cartilage hinge. In addition, Ultrasound is also helpful to assess the entrapped annular ligament injuries in the subluxated fresh Monteggia fracture and pulled elbow.

With the development of surgical techniques and assist tools, ultrasound-guided closed reduction of articular fracture has also achieved good results and has become popular for TRASH-PS fractures.^[23–28]^ Non-articular surface fractures, such as transphyseal separation of distal humerus, radial head osteochondral separation, and Monteggia fracture, could be well treated by ultrasound-guided treatment. It is contraindicated to apply a fixation screw across the growth plate to reduce the risk of premature closure.^[29]^ The method of reducing the annular ligament of a fresh Monteggia fracture is traction of the forearm, supination, and extreme flexion of the elbow joint. If a “click” sound is heard or the “double-hump sign” and “congruency sign” of a radio-capitellum joint are found via ultrasound, these could indicate that the entrapped annular ligament is reduced.^[30]^

## 4. Conclusions

The TRASH-PS fractures are easily misdiagnosed with un-ossification on radiographs and may lead to limb deformity or dysfunction. X-ray is limited in the diagnosis of these fractures. In addition, ultrasound is a radiation-free technique which not only dynamically visualizes the fracture alignment and cartilage, but also could detect ligament damage and monitor reduction process. Thus, ultrasound is recommended in diagnosis and treatment of TRASH-PS fractures. In conclusion, particular care should be taken during the initial physical examination, and an ultrasound examination is advised in highly suspicious TRASH-PS fractures.

## Author contributions

**Conceptualization:** Guoqiang Jia.

**Supervision:** Guoqiang Jia, Jun Sun.

**Writing – original draft:** Guoqiang Jia.

**Writing – review & editing:** Guoqiang Jia.
